# Intramuscular myxoma of the left thigh: A case report

**DOI:** 10.1016/j.ijscr.2022.107710

**Published:** 2022-09-30

**Authors:** Ali Al Abdulsalam, Sarah Al Safi, Sami Aldaoud, Nimer Al-Shadidi, Piyaray Mohan Dhar

**Affiliations:** a Department of General Surgery, Al-Adan Hospital, Hadiya 46969, Kuwait; b Histopathology Department, Al-Adan Hospital, Hadiya 46969, Kuwait

**Keywords:** Intramuscular, Myxoma, Benign, Magnetic resonance imaging, Excision

## Abstract

**Introduction and clinical importance:**

Intramuscular myxoma (IM) is a rare benign soft tissue tumor that involves the musculoskeletal system with a reported incidence of 0.1–0.13 per 100,000. The mean age of diagnosis is 40–70 years of age, with female predilection. The most common site of involvement is the thigh, however, it may present in other areas such as the upper arm, calf, and buttock.

**Case presentation:**

A case of a 45-year-old female without a significant past medical or surgical history who presented with 3-year history of a slow-growing, painless mass in her left upper thigh. MRI scan was performed which showed intramuscular soft tissue mass lesion 9 × 6 × 4.5 cm implicating the left distal vastus medialis muscle. A fine needle aspiration was inconclusive so a core needle biopsy was performed which was suggestive of intramuscular myxoma. A complete surgical excision of the mass was done and the postoperative period was uneventful and patient was discharged home. The final histopathological examination confirmed the diagnosis of intramuscular myxoma.

**Discussion:**

Intramuscular myxoma is a rare benign soft tissue neoplasm. 50 % of cases commonly occur in the thigh. IM has an unknown etiology, however, the literature has showed common gene mutations such as the GNAS gene mutations (Guanine nucleotide binding protein, alpha stimulating). Imaging modalities such as ultrasound, computed tomography (CT) and magnetic resonance imaging (MRI) are useful in diagnosis of soft tissue mass but not specific to intramuscular myxoma. Histopathological examination is the gold standard for diagnosis. The treatment of choice is surgical excision with clear margins to prevent recurrence, which is extremely rare.

**Conclusion:**

Intramuscular myxomas, although benign and rare, should be in the differential diagnosis of soft tissue lesions due to the similarity they share with malignant tumors such as sarcomas. Histopathological examination is the gold standard for diagnosing a soft tissue lesion and surgical excision is the treatment of choice.

## Highlights

• Soft tissue masses are common but intramuscular myxoma is a rare differential diagnosis
• Clinical examination and imaging modalities are inconclusive in formulating the diagnosis of intramuscular myxoma
• Histopathological examination is the gold standard for diagnosing cases of intramuscular myxoma
• Surgical excision with a gross negative margin is the treatment of choice

## 1 Introduction

Intramuscular myxoma (IM) is a rare benign soft tissue tumor that involves the musculoskeletal system with a reported incidence of 0.1–0.13 per 100,000 [1]. The mean age of diagnosis is 40–70 with the majority (57 %) being females [2], [3]. IM mostly presents as a solitary lesion. However, in 5 % of cases, it presents as multiple lesions [4], [5]. The most common location of IM is in the thigh (51 %). However, it can occur in other locations such as the upper arm, calf, and buttock [6]. Surgical excision is the definitive treatment of IM and recurrence is very rare [1]. We present a case of a 45-year-old female who presented with IM of the left thigh. The case is presented in line with the Surgical CAse REport (SCARE) criteria [7].

## 2 Case report

A previously healthy 45-year-old female presented to our hospital from the outpatient clinic with a 3-year history of a slowly growing mass on the anterior aspect of her left mid-thigh which was painless and only caused her slight discomfort. She has a family history of hypertension, type 2 diabetes mellitus and dyslipidemia. She is a non-smoker and non-alcohol consumer. She did not a have a significant drug history nor drug allergies. On examination her vital signs were within normal range. There was a palpable 10 × 10 cm non-tender mass on the anterior left thigh which was solid and had regular borders. The mass did not transilluminate and was not attached to overlying skin.

**Fig. 1:**
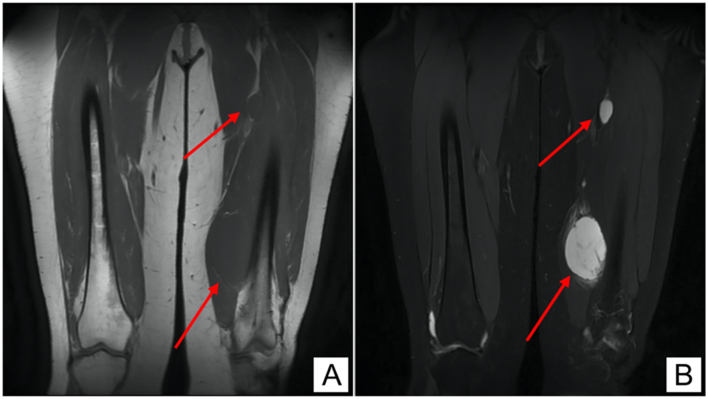
(A) Coronal MRI section showing low signal intensity on T1W imaging in two soft tissue lesions. (B) Coronal MRI section showing high signal intensity on T2W imaging in two soft tissue lesions.

**Fig. 2:**
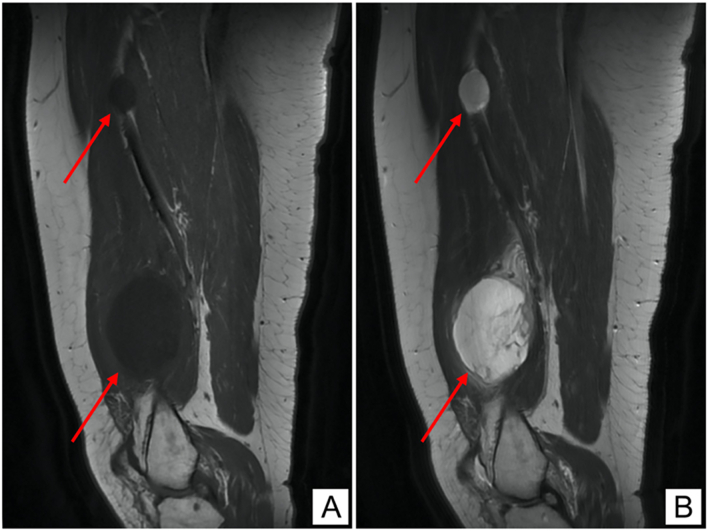
(A) Sagittal MRI section showing low signal intensity on T1W imaging in two soft-tissue lesions. (B) Sagittal MRI section showing high signal intensity on T2W imaging in two soft tissue lesions.

**Fig. 3:**
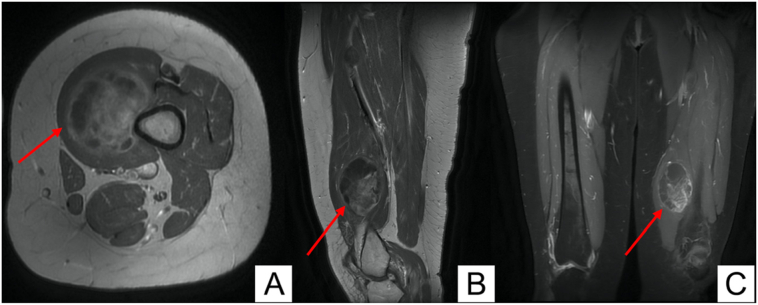
Heterogeneous T1W post-contrast filling in Axial (A), Sagittal (B), and Coronal (C) MRI sections.

The initial laboratory investigations were unremarkable. She then underwent left thigh magnetic resonance scan (MRI) which revealed a globular shaped intramuscular soft tissue mass lesion 9 × 6 × 4.5 cm implicating the left distal vastus medialis muscle. The lesion demonstrated homogenous low T1 (Fig. 1A) and high T2 (Fig. 1B) signals with marked heterogenous post contrast enhancement (Fig. 3A-C). There was another smaller 3 × 2 cm intramuscular lesion with the same criteria noted between the quadriceps and adductor muscles (Fig. 1, Fig. 2).

**Fig. 4:**
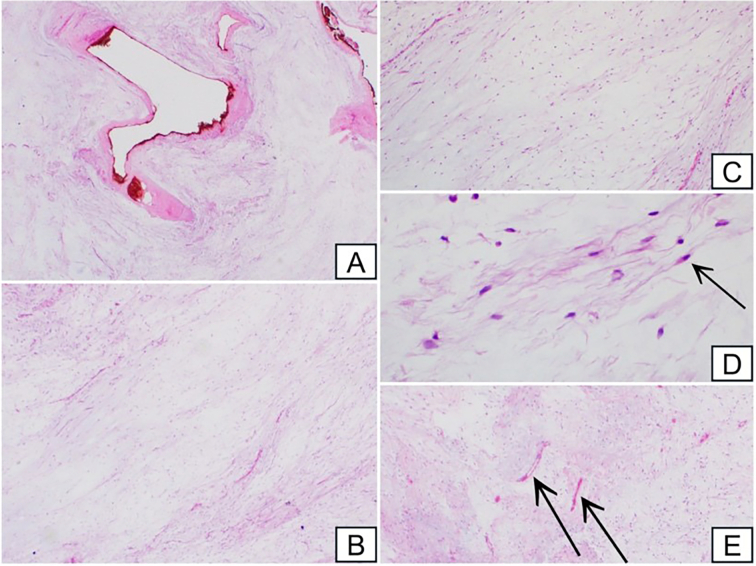
Histopathological image showing lesion with myxoid background (A). Lesion is hypocellular (B). Lesion consists of bland oval two spindle cells, there is no mitosis, and background is myxoid (C-D). Few thin-walled blood vessels (E).

Following that she underwent fine needle aspiration which showed only benign spindle cell lesion. It was followed by core needle biopsy which showed stellate cells that are positive for CD34 and SMA. These cells were negative for PanCk, S100 and Desmin. The final impression was suggestive of intramuscular myxoma.

Subsequently, she was admitted electively and underwent excision of the left thigh mass under general anesthesia. Preoperatively, the patient was sent to radiology department for metallic marker application within the smaller mass for easier intraoperative identification.

An incision was made on the left anterior thigh 10 cm from the left knee, dissection of layers was done until reaching the mass. The mass was dissected from overlying muscles and released from dense attachments bluntly and using electrocautery. Furthermore, a C-arm machine was used intraoperatively to locate the smaller mass that was preoperatively marked. A skin incision overlying the identified site was created and similar steps as described earlier were followed until the mass was completely excised. There were no complications post-operatively, the patient was able to ambulate on the same day with minimal pain at the surgical sites and was discharged on post-operative day two with surgical outpatient follow up. The patient presented to our surgical outpatient clinic 2 weeks later complaining of minimal swelling at the knee joint with limited knee mobility. She was advised referred to orthopedic outpatient clinic for further evaluation. Otherwise the wound healed well with no signs of hematoma or infection. The final histopathological examination revealed a benign lesion consisting of bland cells and basophilic matrix showing few spindle cells. The lesion was capsulated by a collagenized capsule and shows bundles of striated muscle fibers at the periphery (Fig. 4). Immunohistochemistry test was positive for Vimentin and CD34, positive for Alcian blue and Mucicarmine in mucoid matrix, and negative for S100 and PanCk.

## 3 Discussion

The term myxoma was first used by Virehow in 1871 to define a tumor that resembled the mucinous substance in the umbilical cord [1]. Myxoma are a group of mesenchymal tumors that consist of an abundant extracellular myxoid matrix [8]. Benign myxomas, based on radiological and histopathological features are divided into intramuscular myxoma, superficial angiomyxoma, aggressive angiomyxoma, myxolipoma, acral fibromyxoma and dermal myxoma. The most common type of benign myxomas are intramuscular myxomas [9].

Intramuscular myxoma (IM) is a rare benign soft-tissue neoplasm that commonly involves the thigh (51 %). However, it may involve other areas such as the upper arm (9 %), calf (7 %), and buttock (7 %) [6]. IM has a reported incidence of 0.1–0.13 per 100,000 and mean age of diagnosis is 40–70 with the majority (57 %) being females [1], [2], [3]. Although IM has an unknown etiology, it has been demonstrated in the literature that GNAS (guanine nucleotide binding protein, alpha stimulating) gene mutations are common in IMs [5], [10], [11]. Furthermore, new molecular techniques are in development to aid the detection of GNAS gene mutations such as the TaqMan and single-molecule tagged molecular inversion probes (smMIP). The TaqMan is a gene expression assay that uses real time polymerase chain reaction (PCR) expression study, whereas the smMIP is also a sequencing method that is used to detect genetic variations. In a recent article, it has been demonstrated that both the TaqMan and smMIP are crucial and reliable tools in the detection of GNAS gene mutation in IM [12].

Clinically, they may occur as solitary lesions, in which case they may be associated with fibrous dysplasia or McCune-Albright Syndrome [1]. Whereas multiple myxomas in conjunction with fibrous dysplasia are associated with Mazabraud's syndrome [6]. IMs usually present as slow growing, painless, and palpable masses as in our case [13].

Radiographs in IM may be normal or represent a non-specific soft-tissue mass [14]. Ultrasound of IM shows a hypoechoic mass with occasional cystic components [1]. On computed tomography (CT) scan, it shows as a well-circumscribed soft tissue mass with higher attenuation than that of water and less than the surrounding muscles [14]. Furthermore, IM typically demonstrates a low signal intensity lesion on T1-weighted (T1W) images and high signal intensity on T2-weighted (T2W) images on MRI [13]. MRI is superior in characterization of IM MRI images obtained in our case report are in line with the typical presentation of IM on MRI.

Although imaging modalities such as X-ray, CT and MRI play a role in the workup of IM to differentiate it from other malignant lesions such as myxoid chondrosarcoma, myxoid liposarcoma, a definitive diagnosis of IM can be made only by histological examination with core needle biopsy [1]. Typical histopathological appearance of IM includes a lesion with abundant myxoid extracellular matrix. The lesion is usually hypovascular, hypocellular, and contains bland spindle cells [13], [15], [16]. On immunohistochemistry staining, IM usually stain for vimentin and CD34 and are negative for S100 [1]. This is consistent with the immunohistochemistry staining findings in our case. Low grade fibromyxoid sarcoma (LGFMS) often mimics IM. They both have similar histopathological features such as having myxoid background, bland oval spindle cells, and elongated bloods vessels. In recent literature, Mucin 4 (MUC4), which is a protein coding gene that was found in various carcinomas, has been found to be highly sensitive and specific in LGFMS. Absence of MUC4 expression on immunohistochemistry staining is useful in distinguishing IM from LGFMS [17], [18].

The treatment of choice in IM is surgical excision with clear margins. Inadequate excision may lead to local recurrence however, this is extremely rare. Therefore, adequate surgical excision is vital to prevent recurrence [1], [13], [14].

## 4 Conclusion

Intramuscular myxomas, although benign and rare, should be in the differential diagnosis of soft tissue lesions due to the similarity they share with malignant tumors such as sarcomas. A definitive diagnosis of intramuscular myxoma can only be made with histopathological examination. Radical surgical excision is the gold standard treatment, and local recurrence is rare with adequate resection.

## Provenance and peer review

Not commissioned, externally peer-reviewed.

## Consent

Written informed consent was obtained from the patients for publication of this case report and accompanying images. A copy of the written consent is available by the Editor-in-Chief of this journal upon requests.

## Funding

None.

## Ethical approval

The study type is exempted from ethical approval at our institution: case report.

## Author contribution

AA: conducted literature review, drafted the manuscript. SS: conceptualized and critically revised the manuscript. PD, NS: performed the intervention and supervised the case report. SA: Provided histopathology slides and diagnosis. All Authors approved the final manuscript as submitted.

## Guarantor

Sarah H. Al Safi.

## Research registration number

Not applicable.

## Declaration of competing interest

None.
